# Editorial: Biosocial determinants and solutions of mental health conditions in low and middle-income countries

**DOI:** 10.3389/fpubh.2025.1691121

**Published:** 2025-09-12

**Authors:** Rajiv Chowdhury, Natalia Trujillo, Samaun Khalid

**Affiliations:** Robert F Stempel College of Public Health and Social Work, Florida International University, Miami, FL, United States

**Keywords:** global mental health, low and middle income countries, depression, anxiety, suicide

## Introduction

Mental health conditions represent a significant and growing public health challenge, especially in low- and middle-income countries (LMICs). These settings are characterized by numerous factors that foster social inequities and vulnerabilities, which increase the burden of mental disorders (1) However, public mental health spending in LMICs averages only 2.1% of total health budgets (2), limiting the capacity to develop detection, prevention, and care programs to address the rapidly expanding mental health issues.

Mental health conditions often develop early in life, affect people throughout their lives, and are heavily influenced by poverty, gender inequality, and exposure to violence. Depression and anxiety are the leading contributors to the global burden of mental disorders (3). Both are usually linked to unemployment, financial struggles, food and job insecurity, poor housing conditions, exposure to violence, and discrimination (4). They are associated with long-term disability and the risk of suicide, which is a major cause of death among young people worldwide. Therefore, they pose a significant public health challenge and require a stronger health system, investment in community-based care, and efforts to address the social factors that lead to mental distress (3).

## Addressing a gap in global evidence

Over the past few years, the COVID-19 pandemic has revealed significant gaps in global mental health research, especially in resource-limited settings. Specifically, the pandemic has increased the prevalence of depression and anxiety symptoms and may have contributed to higher suicide rates worldwide. It has particularly affected the mental health of individuals from lower socio-economic backgrounds and those with limited access to social support and healthcare. Table 1 summarizes recent studies that assess the global psychological impact of the COVID-19 pandemic across various settings. These findings generally indicate a high worldwide prevalence of depression (up to 82%), anxiety (up to 64%), and suicidal ideation (up to 22%) during the pandemic.

**Table 1:** Mental health impacts of the COVID-19 pandemic in LMICs.

**Study author**	**Year published**	**Location**	**Study design**	**Total participants (*n*)**	**Female (%)**	**Study population/age range**	**Depression (%)**	**Anxiety (%)**	**Suicide ideation (%)**
Seedat et al. (5)	2025	South Africa	Cross-sectional survey	1,211	73.6%	University students; (≥18)	28.0%	36.9%	21.80%
Sun et al. (6)	2024	China	Cross-sectional survey	9,858	-	-	47.10%	-	7.80%
Malebari et al. (7)	2024	Saudi Arabia	Cross-sectional survey	728	-	University students, 18–35 years	81.50%	63.60%	-
Hall et al. (8)	2023	China	Cross-sectional survey	3,230	44.30%	Median age 32 (IQR 26–39)	26.10%	20.10%	3.80%
Akingbade et al. (9)	2023	Nigeria	Cross-sectional survey	590	56%	Adults (≥18), 38% ≥30 years	-	55% (combined with depression)	-
Liang et al. (10)	2022	China	Cross-sectional survey	471,672	62.63%	College students (≥18)	-	-	10.7%
Lusida et al. (11)	2022	Indonesia	Cross-sectional survey	608	38.80%	Median 35 yrs	3.60%	14.30%	-
Ibigbami et al. (12)	2022	Nigeria	Cross-sectional survey	434	67.10%	22–64 years (Mean 37.4)	6.70%	3.20%	-
Jiang et al. (13)	2021	Multicountry, South-East Asia	Cross-sectional survey	1,195	67.10%	18–35 years	46.5%	20.5%	-
Rahman et al. (14)	2021	Bangladesh	Cross-sectional survey	1,415	38.20%	18–61 years	-	-	19.0%
Cénat et al. (15)	2021	Multicountry, Sub-Saharan Africa	Cross-sectional survey	1,267	40.9% overall	< 24, 25–34, 35+	24.37%	-	-
Shah et al. (16)	2021	Multicountry	Cross-sectional survey	678	57.2%	All ages	58.6%	50.9%	-

To provide new research evidence from geographically diverse contexts, we previously launched a Research Topic on “*Biosocial determinants and solutions for mental health conditions in low and middle-income countries: revealing the current evidence gaps*”. This Research Topic includes several original studies that explore (a) the roles of social and demographic factors in mental health outcomes, especially during rapid changes like pandemics, armed conflict, and migration, and (b) offer guidance on cultural evidence necessary to shape public mental health initiatives in LMIC.

First, reports published in this Research Topic include an account of how COVID-19 restrictions impacted mental health. In a study with Chinese postgraduate students, Zhang et al. report higher stress and depression scores among students, especially rural female students. These scores were also associated with increased insomnia and suicidal ideation. The report calls for targeted psychological interventions and academic support systems that address students' specific needs. In Ghana, Nyawornota et al. investigate the impact of the COVID-19 pandemic on self-perceived health and physical activity levels among youth and adults. The results show significant sex differences in physical activity and self-perceived health during the pandemic, with women reporting lower activity levels and poorer health. These studies provide (1) a critical understanding of the evolution of health behaviors and perceptions under crisis conditions, (2) the psychosocial implications of being part of the healthcare workforce, and (3) the role of incorporating limited physical activity routines.

Second, several studies on this issue emphasize the importance of culturally sensitive research from Southeast Asia and the Middle East. In Ilam, Iran, Bazyar et al. present findings from a new population-based study that deepens our understanding of subjective health perceptions in low-resource settings. They examine the factors influencing self-rated health (SRH) and its value as a mental health indicator in conflict-affected and socioeconomically disadvantaged areas. The results show that poor SRH was predicted by hopelessness about the future and the presence of underlying chronic diseases. This research offers a scalable, low-cost alternative for identifying mental health issues to guide community health planning and policy in similar global contexts. In Northwest China, Niu and Wang conducted a study to understand better the psychological wellbeing of rural left-behind women (RLW). These women face significant challenges related to being the household head and raising children in vulnerable rural environments while their husbands migrate to urban areas for work. The study found that 35.7% of participants reported symptoms of depression, 37.6% experienced anxiety, and felt less secure. Notably, this study emphasizes the importance of integrating gender-sensitive approaches to assess the mental health impact of internal migration in similar LMIC settings. In Nepal, Mamidanna et al. explored the mental health effects of widowhood among women in a region where widowhood carries stigma and social isolation. The study revealed that widowed women exhibited high levels of emotional distress, anxiety, and depression, which could be worsened by age, poverty, and household income. These three studies highlight the critical intersection of gender, socioeconomic vulnerability, and cultural norms in societies that often experience social exclusion and economic hardship.

Third, studies in this issue highlight a significant, but often overlooked, role of environmental and economic stressors in impacting mental health outcomes. For example, during prolonged political and financial crises, El Khoury-Malhame et al. present new data from Lebanon showing that maladaptive coping strategies and intolerance of uncertainty are strongly linked to anxiety, depression, reduced wellbeing, and greater symptom expression among women. In contrast, adaptive coping strategies and social support are associated with better mental health outcomes. Additionally, in a longitudinal study in China, Liu and Zhang examine the psychological effects of household debt and find that indebtedness considerably worsens mental health. These studies underscore the importance of including mental health interventions that address structural (income, armed conflict) and relational (support, coping strategies) factors, especially in LMICs where financial insecurity is common.

Finally, since measurement gaps continue to be a major barrier to timely mental health assessment, Díaz-Castro et al. seek to improve pediatric mental health evaluation by validating the World Health Organization's Disability Assessment Schedule (2.0) for use with children and adolescents with mental disorders in specialized psychiatric care settings in Mexico. This study emphasizes the need for culturally appropriate assessment tools and scalable interventions.

In conclusion, this Research Topic presents new evidence on biosocial determinants of mental health in LMICs and highlights an unmet need for a coordinated, multifaceted approach. The studies will remind readers that (a) mental health must be incorporated into global health strategies, (b) such strategies should be developed through effective transdisciplinary collaboration supported by targeted funding, and (c) careful contextual implementation and community cohesion are crucial in addressing the rising burden of mental health issues worldwide.
